# Assessment of dental caries and salivary nitric oxide levels in children with dyspepsia

**DOI:** 10.1186/s12903-018-0707-z

**Published:** 2019-01-11

**Authors:** Damla Aksit-Bicak, Ebru Emekli-Alturfan, Unsal Veli Ustundag, Serap Akyuz

**Affiliations:** 1 Faculty of Dentistry, Department of Pediatric Dentistry, Near East University, Near East Boulevard, ZIP: 99138 Mersin 10, Turkey; 2 Faculty of Dentistry, Department of Basic Medical Sciences, Biochemistry, Marmara University, Istanbul, Turkey; 3 Faculty of Medicine, Department of Basic Medical Sciences, Biochemistry, Medipol University, Istanbul, Turkey; 4 Faculty of Dentistry, Department of Pediatric Dentistry, Marmara University, Istanbul, Turkey

**Keywords:** Nitric oxide, Saliva, Dental caries, Dyspepsia, Gastric diseases

## Abstract

**Background:**

The increase in nitric oxide (NO) levels in the oral cavity and saliva have been associated with various oral diseases; however, the gastro-salivary interaction of NO remains controversial. Thus, the aim of this study was to determine and compare salivary NO levels of dyspeptic and non-dyspeptic healthy children and to conduct an evaluation of its association with dental caries.

**Methods:**

Seventy children with dyspepsia (dyspeptic group) and 30 children without any gastrointestinal complaints (control group) were included in the study. Two biopsies from the gastric tissues were collected from dyspeptic children for histopathologic examination. Oral examination involved the assessment of dental caries, gingival index, plaque index, buffering capacity, salivary flow rate and pH. Salivary *Streptococcus mutans (S. mutans) and Lactobacilli sp.* counts were performed by commercial kits. For the comparison of the normal distribution between dyspeptic and control groups, Student t-test and for the comparison of the non-normal distribution, Kruskal-Wallis and Mann-Whitney-U tests were used. Chi-square test was used for comparison of qualitative data and the Pearson correlation test was used to evaluate the association between certain variables. Significance was assessed at *p* < 0.05 level.

**Results:**

*Helicobacter pylori* (*H.pylori*) were found in gastric biopsies of 84.2% (59/70) of the dyspeptic children. While the mean salivary NO values did not differ significantly between gastric *H.pylori* positive, negative and control groups, the salivary NO level of the dyspeptic group (213.7 ± 51.68 μmol/dL) was found to be significantly higher than the control group (185.7 ± 16.66 μmol/dL). No significant relationship was found between the mean salivary NO values, DMFT/dmft numbers and other oral parameters.

**Conclusions:**

The association of dental caries and salivary NO levels could not be considered specific in the current study. Although there were no statistically significant differences between salivary NO levels of gastric *H.pylori* positive, gastric *H.pylori* negative and control groups, greater salivary NO levels among dyspeptic children compared with the control group demonstrated that the concentration of NO in the saliva could be used as a biological marker in dyspepsia, which could lead to the improvement of more specified, uncomplicated and susceptible methods for analysis.

## Background

Nitric oxide (NO) is a free radical with a gas structure that is created from an extensive diversity of cells and tissues in the individuals’ body. It can be diffused easily from the membranes and is also included in the adjustment of numerous physiological activities, such as cell death, immune regulation, neurotransmission, and vascular relaxation. Additionally, remarkable differences have been detected in the levels and effects of NO in each oral cavity tissue [1]. Although other free radicals are harmful to cells at every concentration, NO at low concentrations has a role in important physiologic functions. However, excessive and uncontrolled NO synthesis is detrimental to cells [2, 3]. Nitric oxide, with these properties, is an ideal physiologic signaling molecule [4, 5]. In 1987, nitric oxide synthase (NOS) was discovered during the isolation of vascular endothelium from a structure known as endothelium-derived relaxation factor (EDRF), and the EDRF was found to be NO in the following years [6, 7].

Dyspepsia is characterized by fullness, bloating, nausea, recurrent subjective upper abdominal discomfort or epigastric pain. In spite of dyspeptic symptoms, if there are no structural and biochemical changes that may clarify the symptoms, then the disease is called functional or non-ulcer dyspepsia. In this case, symptoms may be caused by changes in the secretion, sensitivity or motility of the alimentary canal [8]. It is comprehended that NO contributes to the regulation of gastric mucosal integrity and the control of gastric emptying. It has been shown that high concentrations of NO in the gastric lumen, leads gastric relaxation and causes relief of functional dyspepsia symptoms, while it also has a crucial role in protecting the stomach from hazardous pathogens [8–10].

The initial part of the digestive system is the oral cavity, where food and other secretions pass through to the rectum. The oral flora form the most complex microbial community of the human body, and consist of more than 700 types of bacteria, viruses, and fungi [11]. Saliva has a crucial role in the host defense mechanism by including several specific and nonspecific defense factors. As soon as nitrate within the saliva is secreted into the oral cavity, it is rapidly converted into NO by oral flora and salivary peroxidase. Consumption of nitrate containing vegetables may have a crucial role in the defense of the oral cavity and stomach from pathogenic microorganisms via the gastrointestinal–salivary cycle of NO and its metabolites [12–14]. Thus, salivary NO levels might reflect gastric diseases, such as dyspepsia via the gastrointestinal–salivary cycle of NO.

Increased NO levels in the oral cavity and saliva for various reasons have been associated with oral diseases [1]. A relationship has been demonstrated between increased salivary NO levels and several oral diseases, particularly dental caries [15–17]. In the determination of the role of NO on caries formation, it is suggested to examine other salivary parameters related to caries development such as pH and salivary flow rate [18].

Therefore, the intention of this study was to determine and compare salivary NO levels of dyspeptic and non-dyspeptic healthy children and to conduct an evaluation of its association with dental caries. The null hypothesis was that there is no difference between salivary NO levels of dyspeptic and control groups, and there is no relationship between the development of dental caries and salivary NO levels.

## Methods

### Ethical approval and selection of subjects

Ethical approval was obtained from the Marmara University, Institute of Health Sciences Non-invasive Clinical Research Studies Ethics Committee (24.12.2014–13). Parents of all children signed informed consent prior to the sample collection process.

The studied population comprised 70 dyspeptic (dyspeptic group) and 30 non-dyspeptic children (control group) all of whom were 6–16 years old. The majority of the studied population of the current study was composed of children from our previous study [19] apart from four dyspeptic children who did not want to continue, which meant that four dyspeptic individuals were included in their place. Endoscopy was performed in the dyspeptic group. All children in the control group were healthy without any systemic diseases until the time of examination. The exclusion criteria for both groups included previous eradication therapy and not volunteering to participate [19].

### Questionnaire

With a questionnaire, medical history, demographic data, and oral hygiene habits were noted.

### Oral examination and collection of saliva and gastric specimens

Oral examination was conducted by one specialist using a disposable dental mirror under a portable light. Dental caries was diagnosed according to the World Health Organization 1997 criteria [20]. The plaque index and gingival index were determined according to Silness and Löe [21] and Löe and Silness [22], respectively.

All children were asked not to consume anything 2 h before the collection of saliva. In addition, they should not have used any mouthwash during that period. The saliva collection was performed during morning hours for both groups and before the endoscopy procedure for the dyspeptic group. The children were instructed to spit into a funnel over a 15 ml test tube until a sample volume of 2 ml of saliva could be accumulated. After the collection procedure, the specimens were stored at − 20 °C until used for total NO determination. All 70 dyspeptic patients’ parents agreed to the collection of 2 biopsies from the gastric tissues for histopathologic examination [19].

### Assessment of the buffering capacity, salivary flow rate, pH and the levels of cariogenic bacteria

Buffering capacity was determined with the Ericsson method and salivary pH was evaluated with a pH meter (Thermo Scientific™, USA) from all participants [19]. For the assessment of stimulated salivary flow rates, masticatory stimulation was induced by chewing paraffin wax gum, and the subjects were requested to spit for 5 min into a funnel inserted into a vial.

A chair-side method was used to measure the levels of *Streptococcus mutans* (*S.mutans*) and *Lactobacilli* in saliva samples (Dentocult SM and LB kit, Orion Diagnostica, Espoo, Finland) according to the producer’s directions [19, 23].

### Salivary NO assay

Salivary NO levels were determined according to the method of Miranda et al. [24]. The fundamental of the assay was the reduction of nitrate by vanadium (III) united with evaluation by the acidic Griess reaction. Griess reagents were prepared as separate solutions of N-(1-Naphthyl)-ethylenediamine dihydrochloride (0.1% wt/vol) in H_2_0 and sulfanilamide (2% wt/vol) in 5% HCl. The absorbances were measured spectrophotometrically at 540 nm. The results were assessed using the extinction coefficient 53,000 M^− 1^/cm^− 1^ and expressed as μmol % [24].

### Statistical analysis

Statistical analyses of all data were performed using the SPSS 15 package programme (SPSS, Inc., Chicago, IL, USA). For the comparison of the normal distribution between dyspeptic and control groups, Student t-test and for the comparison of the non-normal distribution, Kruskal-Wallis and Mann-Whitney-U tests were used. Chi-square test was used for comparison of qualitative data and the Pearson correlation test was used to evaluate the association between certain variables. Significance was assessed at *p* < 0.05 level.

The sample size was calculated using power analysis prior to the study. With 73% targeted power and 0.05 confidence level, using the previous literature, it was necessary for 97 individuals to be enrolled in this study. To ensure statistical power, a total of 100 children were included.

## Results

The study comprised 100 children aged 6–16 years (mean age = 10.12 ± 3.14. years, mean age for the dyspeptic group = 11.23 ± 3.44 years and mean age for the control group = 7.87 ± 1.99) (*p* < 0.05). In the dyspeptic group, there were 41 (58%) girls and 29 (41%) boys. In the control group, there were 12 (40%) girls and 18 (60%) boys (Table 1).

**Table 1:** Distribution of the participants by age and gender

		Dyspeptic Group (*n* = 70)	Control Group (*n* = 30)	
Age	Mean	11,23	7,87	p
	Standart Deviation (SD)	3,44	1,99	< 0.0001^1^
Gender	Boys	29	18	^2^0.088
	Girls	41	12	

^1^Mann Whitney U test ^2^Chi-square test

All children in the control group were healthy without any systemic diseases. Children in the dyspeptic group had no other systemic diseases except for dyspeptic symptoms. The most common symptom was epigastric pain in 61 (61%) patients, followed by nausea (41%), reflux (38%), regurgitation/vomiting (27%), constipation (24%) and diarrhea (17%).

*Helicobacter pylori* (*H.pylori*) were examined in the gastric biopsies of 84.2% (59/70) of the children with dyspepsia. The mean salivary NO values were not statistically significant between gastric *H.pylori* positive, negative and control groups (χ^2^ = 0.573, *p* = 0.751) (Table 2). The mean salivary NO value for the dyspeptic group was 213.7 ± 51.68 μmol/dL and 185.7 ± 16.66 μmol/dL for the control group. After applying the t-test, the salivary NO level of the dyspeptic group was found to be higher significantly than the control group (*p* = 0.0046) (Table 3). No significant relationship was found between the ages of the participants and the mean salivary NO values (*r* = 0.075, *p* = 0.461).

**Table 2:** The mean salivary nitric oxide values of gastric *H.pylori* positive, negative and control groups

		N	Mean	Std. Deviation	Minimum	Maximum
Dyspeptic Group (*n* = 70)	Gastric H.pylori (−)	11	207,6417	46,34,549	170,00	298,00
	Gastric H.pylori (+)	59	214,9655	53,02861	170,00	296,00
	Control Group (*n* = 30)	30	185,6700	16,66,272	156,00	231,10
	Total	100	205,2980	45,94,782	156,00	298,00

Kruskal Wallis test (χ^2^ = 0.573, *p* = 0.751)

**Table 3:** The mean salivary nitric oxide values, DMFT/dmft index, the gingival index, the plaque index, the salivary flow rate, pH and the buffering capacity of dyspeptic and control groups

	Dyspeptic Group (*n* = 70)		Control Group (*n* = 30)		
	Mean	Standard Deviation (SD)	Mean	Standart Deviation (SD)	p
NO	213.7	51.68	185.7	16.66	^1^0.0046
DMFT	5,18	4,14	2,19	2,14	< ^2^0.0001
dmft	3,29	2,79	7,41	4,68	^2^0.001
Gingival index	0,29	0,54	0,04	0,09	^2^0.039
Plaque index	0,76	0,58	0,34	0,30	< ^1^0.0001
Salivary flow rate	6,29	2,77	5,85	2,17	^1^0.439
pH	7,44	0,33	7,61	0,38	^1^0.048
Buffering capacity	5,93	0,39	5,98	0,49	^1^0.649

*NO* Nitric Oxide, *DMFT* decayed, missing and filled permanent teeth; *dmft* decayed, missing, and filled primary teeth

^1^Student t test ^2^Mann Whitney U test

Amongst the dyspeptic group, the mean DMFT numbers, the gingival index, and the plaque index scores were found to be higher and the mean dmft numbers and pH values were found to be lower compared with those for the control group (*p* < 0.05) (Table 3). In addition, a weak correlation was determined between the ages and gingival index (*r* = 0.263, *p* = 0.008), plaque index (*r* = 0.254, *p* = 0.011) and pH values (*r* = − 0.281, *p* = 0.005).

The effect of variables like buffering capacity and the salivary flow rate was found to be statistically not significant between the dyspeptic and control groups (*p* > 0.05) (Table 3). A weak correlation was determined between the age and salivary flow rate (*r* = 0.397, *p* = 0.01). No significant relationship was found between the ages and buffering capacity (*r* = − 0.007, *p* = 0.947).

Additionally, no significant relationship was found between the mean salivary NO values and other variables such as DMFT numbers (*r* = 0.040, *p* = 0.703), gingival index (*r* = 0.017, *p* = 0.870), plaque index (*r* = 0.044, *p* = 0.666), salivary flow rate (*r* = 0.087, *p* = 0.389), pH (*r* = − 0.036, *p* = 0.726) and buffering capacity (*r* = − 0.044, *p* = 0.665) according to Pearson’s correlation test. A very weak, negative correlation was determined between salivary NO levels and dmft numbers (*r* = − 0.240, *p* = 0.063).

The frequency of daily toothbrushing, daily sugar intake, *Streptococcus mutans* (χ^2^ = 2.606, *p* = 0.456) and *Lactobacilli* sp. numbers (χ^2^ = 1.018, *p* = 0.907) (Table 4) did not differ significantly between dyspeptic and control groups (*p* > 0.05).

**Table 4:** Streptococcus Mutans and Lactobacilli counts of dyspeptic and control groups

	Streptococcus Mutans					Lactobacilli					
	< 10.000 CFU/ml	10.000–100.000 CFU/ml	100.000–1.000.000 CFU/ml	> 1.000.000 CFU/ml	Total	< 1000 CFU/ml	1000 CFU/ml	10.000 CFU/ml	100.000 CFU/ml	1.000.000 CFU/ml	Total
Dyspeptic Group (*n* = 70)	5	10	13	2	30	5	4	8	7	6	30
	16,7%	33,3%	43,3%	6,7%	100,0%	16,7%	13,3%	26,7%	23,3%	20,0%	100,0%
	50,0%	32,3%	25,0%	28,6%	30,0%	23,8%	26,7%	36,4%	33,3%	28,6%	30,0%
Control Group (*n* = 30)	5	21	39	5	70	16	11	14	14	15	70
	7,1%	30,0%	55,7%	7,1%	100,0%	22,9%	15,7%	20,0%	20,0%	21,4%	100,0%
	50,0%	67,7%	75,0%	71,4%	70,0%	76,2%	73,3%	63,6%	66,7%	71,4%	70,0%

Chi-square test for streptococcus mutans counts (χ^2^ = 2.606, *p* = 0.456) Chi-square test for Lactobacilli counts (χ^2^ = 1.018, *p* = 0.907)

## Discussion

The entrance to the digestive system is the oral cavity, which reflects an individual’s general health conditions and particularly demonstrates disorders of the digestive system. *Helicobacter pylori*, which is a gram-negative, spiral or curve formed and motile bacteria, is responsible for many gastrointestinal disorders [25]. A recent study reported that excretion of NO in exhaled breath was markedly changed in *H. pylori*-infected nonulcerative dyspepsia and peptic ulcer patients. In the same study, molecular NO in exhaled breath was suggested to be used as a potential biomarker for noninvasive diagnosis and selective differentiation of nonulcerative dyspepsia and peptic ulcer diseases. Also, active uptake of nitrate from the bloodstream occurs in the salivary glands [26–29]. Therefore, we aimed to determine whether salivary NO levels could be a potential biomarker for noninvasive diagnosis of dyspepsia and gastric *H.pylori* infection. Although greater salivary NO levels were found among dyspeptic children when compared with the control group, we failed to demonstrate a significant difference of the mean salivary NO values in the presence of gastric *H.pylori* infection. Thus, according to these results, NO in the saliva could potentially be used as a biomarker in dyspepsia, but not in the identification of gastric *H.pylori* infection.

Caries is a multifactorial infectious oral disease, and salivary components secreted in saliva are important for maintaining oral health. The innate defense factors identified in saliva can manage cariogenic bacteria and may prevent the subsequent development of dental caries [30]. Although other components of saliva have crucial roles in the management of caries, the beneficial antimicrobial role of NO in the oral cavity has been a remarkable issue in recent years [31]. Dental caries occurs as a result of a complex interaction between acidogenic microorganisms, fermentable carbohydrates, and host factors including teeth and saliva. Thus, while investigating the antimicrobial role of NO in the oral cavity [32], we evaluated all these factors including buffering capacity, salivary flow rate, pH, *S.mutans* and *Lactobacilli* levels of saliva, plaque and gingival index, oral hygiene and the nutritional habits of individuals in order to demonstrate the effect of salivary NO on caries development that is different from previous studies.

Conflicting results exist regarding the relationship between caries and salivary NO based on DMFT/dmft indices. Eagappan et al. [33] reported that the concentration of NO and its metabolites in the saliva of children with healthy natural teeth was significantly higher compared with that found in the early childhood caries group. Other studies have also reported an inverse correlation between dental caries and salivary NO levels of children. According to these results, the researchers supported the antimicrobial activity of salivary NO in the oral cavity [15, 18, 26, 34–36]. On the contrary, there were studies [17, 37–40] that have found increased NO levels and increased DMFT/dmft indices. These studies supported the defense mechanism of salivary NO associated with the increase in dental caries. These researchers accepted the assumption that the increase in the bacterial quantity according to increased DMFT might have occurred in the increased formation of NO by nitrate reductase enzyme synthesized by facultative anaerobic bacteria when revealed to a hypoxic condition, allowing the nitrite ion to be metabolized [17, 37–40]. In the present study, the dyspeptic group was found to have higher DMFT scores than the control group, however, the control group was found to have higher dmft scores when compared with the dyspeptic group. After applying correlation tests it was shown that these results are mostly related to the difference in the ages of participants and not related to the dyspeptic state or NO concentrations.

The effect of NO on caries formation could not be assessed correctly without evaluating other salivary parameters and environmental conditions [18]. Thus, in this study, other salivary parameters and levels of cariogenic bacteria were also evaluated. Amongst the dyspeptic group, the gingival index and the plaque index scores were found to be higher and pH values were found to be lower compared with those in the control group. The salivary flow rate, buffering capacity, the frequency of daily toothbrushing, daily sugar intake, *Streptococcus mutans*, and *Lactobacilli* numbers did not differ significantly between dyspeptic and control groups. Dong-Hun et al. [41] demonstrated a relationship between salivary *Lactobacilli* levels and salivary NO levels. However, the researchers were unable to exhibit a significant association between *S. mutans* levels and caries activities of subjects participating in their study. In the current study, no significant relationship was found between the mean salivary NO values and other variables such as gingival index, plaque index, buffering capacity, salivary flow rate, and pH. Thus, according to these results, the association of dental caries and salivary NO levels could not be considered specific. The reasons for the differences in the studies might be the differences in the selection of study groups and the methodology used in these studies.

## Strength and limitations

In this study, gingival index, plaque index, buffering capacity, salivary flow rate, pH, and levels of cariogenic bacteria were also determined besides DMFT/dmft indices while evaluating the role of salivary NO on caries formation that is different from previous studies. The limitation of the study is the differences between the studied groups with respect to age and gender.

## Conclusion

In conclusion, to our knowledge, our preliminary study is the first to reveal a possible connection between salivary NO, caries and dyspeptic complaints in children. In this research, we failed to demonstrate a clear relationship between salivary NO levels and dental caries. Thus, further studies are needed to identify the exact role of salivary NO by evaluating other salivary parameters, because dental caries occurs through a complex interaction of many factors.

Identifying the interaction of salivary NO levels with dyspepsia and gastric *H.pylori* infection was another target of this study. Although there were no statistically significant differences between salivary NO levels of gastric *H.pylori* positive, gastric *H.pylori* negative and control groups, greater salivary NO levels among dyspeptic children compared with the control group demonstrated that the concentration of NO in the saliva could be used as a biological marker in dyspepsia, which could lead to the improvement of more specified, uncomplicated and susceptible methods for analysis. Further studies with a larger number of subjects are needed to illuminate the mechanism of gastrosalivary interaction of NO.

## Abbreviations

DMFT/dmft: Decayed, missing or filled teeth
EDRF: Endothelium-derived relaxation factor
*H. pylori*: *Helicobacter pylori*
NO: Nitric oxide.
NOS: Nitric oxide synthetase
*S. Mutans*: *Streptococcus mutans.*
